# Cervix carcinoma is associated with an up-regulation and nuclear localization of the dual-specificity protein phosphatase VHR

**DOI:** 10.1186/1471-2407-8-147

**Published:** 2008-05-27

**Authors:** Rachel Henkens, Philippe Delvenne, Mohammad Arafa, Michel Moutschen, Mustapha Zeddou, Lutz Tautz, Jacques Boniver, Tomas Mustelin, Souad Rahmouni

**Affiliations:** 1 Immunology and Infectious Diseases Unit, GIGA-R, Liège University, Liège, Belgium; 2 Department of Pathology, GIGA-R-CRCE, Liège University, Liège, Belgium; 3 The Burnham Institute for Medical Research, 10109 N Torrey Pines Road, La Jolla, CA 92037, USA

## Abstract

**Background:**

The 21-kDa Vaccinia virus VH1-related (VHR) dual-specific protein phosphatase (encoded by the *DUSP3 *gene) plays a critical role in cell cycle progression and is itself regulated during the cell cycle. We have previously demonstrated using RNA interference that cells lacking VHR arrest in the G1 and G2 phases of the cell cycle and show signs of beginning of cell senescence.

**Methods:**

In this report, we evaluated successfully the expression levels of VHR protein in 62 hysterectomy or conization specimens showing the various (pre) neoplastic cervical epithelial lesions and 35 additional cases of hysterectomy performed for non-cervical pathologies, from patients under 50 years of age. We used a tissue microarray and IHC technique to evaluate the expression of the VHR phosphatase. Immunofluorescence staining under confocal microscopy, Western blotting and RT-PCR methods were used to investigate the localization and expression levels of VHR.

**Results:**

We report that VHR is upregulated in (pre) neoplastic lesions (squamous intraepithelial lesions; SILs) of the uterine cervix mainly in high grade SIL (H-SIL) compared to normal exocervix. In the invasive cancer, VHR is also highly expressed with nuclear localization in the majority of cells compared to normal tissue where VHR is always in the cytoplasm. We also report that this phosphatase is highly expressed in several cervix cancer cell lines such as HeLa, SiHa, CaSki, C33 and HT3 compared to primary keratinocytes. The immunofluorescence technique under confocal microscopy shows that VHR has a cytoplasmic localization in primary keratinocytes, while it localizes in both cytoplasm and nucleus of the cancer cell lines investigated. We report that the up-regulation of this phosphatase is mainly due to its post-translational stabilization in the cancer cell lines compared to primary keratinocytes rather than increases in the transcription of DUSP3 locus.

**Conclusion:**

These results together suggest that VHR can be considered as a new marker for cancer progression in cervix carcinoma and potential new target for anticancer therapy.

## Background

The human genome contains 61 genes for Vaccinia virus H1-like, or 'dual-specific' protein phosphatases (DUSPs) [1], most of which have poorly understood functions. Many of these genes encode phosphatase that dephosphorylate the mitogen-activated protein kinases (e.g. MKP1/*DUSP1*, PAC1/*DUSP2*, MKP3/*DUSP6*, etc) or regulate the cell cycle (e.g. CDC14A and CDC14B). A group of 19 of these phosphatases consists only of a catalytic domain and have molecular weights of 18 – 26 kDa. One of them is the 185-amino acid residues Vaccinia H1-related (VHR), encoded by the *DUSP3 *gene [2], which dephosphorylates and thereby inactivates the mitogen-activated protein kinases Erk and Jnk *in vivo*. We have recently reported that the level of VHR fluctuate during the cell cycle: in early G1, VHR is barely detectable and then it increases to reach a peak before mitosis. Furthermore, the elimination of VHR by RNA interference resulted in cell cycle arrest in G1/S and G2/M [3,4]. This effect of VHR knock-down was counteracted by down-modulation of the levels of Erk and Jnk or by modest levels of Mek and Jnk inhibitors. Based on these data we proposed that VHR is important for cell cycle progression because it tempers Erk and Jnk during the S and G2/M phases of the cell cycle, where excessive activity of these kinases can activate cell cycle check points. The permissive role of VHR in cell cycle regulation prompted us to ask if VHR levels are perturbed in cancer cells.

Uterine cervical cancer is an important health problem [5]. Human papilloma virus (HPV) is strongly implicated as a causative agent in the etiology of this type of cancer [6], which is preceded by well-characterized squamous intraepithelial lesions (SIL), only 10–15% of which will progress to malignancy [7]. However, HPV infection alone is not sufficient to induce cancer development. The role of the immune response in controlling HPV infection and subsequent development of SIL is indirectly established by the increased frequency of HPV-associated lesions in patients with depressed cell-mediated immunity. Indeed, our laboratory has demonstrated that HPV infection is associated with an abnormal cytokine production and diminished APC density function in the normal transformation zone (TZ) where the majority of SIL occurs [8]. How HPV causes premalignant and malignant disease appears to be a consequence of the signaling pathways that are stimulated or repressed in cells to enable the virus to replicate. Major targets of the viral proteins E6, E7 and E5 include respectively p53, proteins encoded by the retinoblastoma gene family and the MAPK activity [9]. Our laboratory has also showed that functional components of the NF-kB signaling pathway are up-regulated and sequestered in the cytoplasm of human papillomavirus 16 (HPV16) transformed cell lines leading to a reduced activity of NF-κB [10]. Mutations in p53 gene have been also reported [11]. It is clear that more investigations on signaling pathways are required to better understand the tumorigenesis associated with HPV infections.

## Methods

### Case selections and microarray construction

Following approval from our institutional review board, a tissue microarray (TMA) was constructed with 62 hysterectomy or conization specimens showing the various (pre) neoplastic cervical epithelial lesions and 35 additional cases of hysterectomy performed for non-cervical pathologies, from patients under 50 years of age. The cases were retrieved from the archived Tissue Bank at Liège University (Liège Biothèque: BTULg) and represented biopsies diagnosed between the years 1998 and 2006.

These cases were selected on the basis of availability of at least one evaluable tissue representative hematoxylin and eosin-stained slide and a paraffin block. For each case, slides were reviewed to select a representative area. The corresponding spot on the associated paraffin block was then cored and placed on a tissue microarrayer (Beecher Instruments, Sun Prairie, WI, USA). The TMA blocks were constructed in doublets (2 spots for each diagnostic entity) using 1 mm tissue cores (Alphelys, Plaisir, France).

The whole series supplied a total number of 194 spots including duplicate of 11 L-SIL, 18 H-SIL, 12 SCC, 12 primary cervical adenocarcinoma (ADC) and 9 adenocarcinoma in situ (AIS). From the cases without cervical lesions, duplicate of 16 ectocervix and 19 endocervix tisues were arrayed. The finalized arrays were then cut into 5 μm-thick sections and mounted on glass slides.

In addition to the formalin-paraffin embedded tissues that were used for the TMA preparation, additional frozen cervical biopsy specimens were retrieved from the Tumor Bank of Liege University (BTULg). These biopsies included 10 high-grade squamous intraepithelial lesions (HSIL), 10 invasive squamous cell carcinoma (SCC) and 10 paired normal exocervical tissues from the same patients. These biopsies were used to perform immunofluorescence studies under confocal microscopy.

### Immunohistochemistry and immunofluorescence

The tissue microarray slides were stained with antibodies against VHR (Clone 237020 dilution 1:2500, R&D Systems, Minneapolis, MN) using a standard avidin-biotin complex method. Tissue microarray slides were deparaffinized with xylene, graded alcohol then rehydrated with distilled water. Endogenous peroxidase activity was blocked by placing the slides in 0.5% hydrogen peroxidase/methanol for 10 minutes followed by a tap water rinse. Background staining was reduced by incubating slides in 0.3% bovine serum albumin/Tris-buffered saline. Antigen retrieval entailed placing the slides in a pressure cooker with an antigen unmasking solution (0.01 M citrate buffer, pH 6.0) for 1 minute. Slides were subsequently incubated with the primary (4°C overnight), then biotinylated secondary antibodies and streptavidin-biotin peroxidase. 3'3' diaminobenzidine (DAB) was used as chromogen and sections were counterstained with hematoxylin.

Immunofluorescence staining on the cervix biopsies was performed with a monoclonal antibody directed against VHR (Clone 24 dilution 1:2500, BD, San Diego, CA). The primary antibodies were revealed with Alexa-488 conjugated secondary antibody together with TOTO-3 to stain nuclei. The sections were mounted and viewed under a confocal laser scanning microscopy TCS SP2 (Leica TCS SP2, Van Hopplynus, Belgium).

For immunofluorescence and immunohistochemistry on cells in culture, the cells were grown on poly-L-lysine coated coverslips and fixed with 4% formaldehyde. The fixed cells were permeabilized with 0.3% of Triton X-100/PBS buffer then stained either with anti-VHR mAb (4 μg/ml) (BD-transduction laboratories) or with p16 antibody (NeoMarkers). After 3 washes, the primary Ab was revealed with an Alexa-488 conjugated secondary Ab together with TOTO-3 (Invitrogen) to stain nuclei and visualized under confocal microscopy. For immunohistochemistry, the anti-VHR antibody was revealed using an HRP secondary detection kit (Universal LSABTM 2 KIT/HRP, Rabbit/Mouse, DakoCytomation). The stained cells were mounted and visualized on light microscopy.

### Scoring of immunohistochemical staining

The VHR immunostaining was scored semi-quantitatively. For staining intensity, 0 represented samples in which VHR nuclear and/or cytoplasmic staining was undetectable, whereas 1+, 2+, and 3+ denoted samples with low, moderate, and strong staining, respectively. For staining extent, in normal ectocervix and the various grades of SIL, 1+ represented samples in which VHR expression was detectable in the lower 1/3 of the epithelium whereas 2+ denoted samples in which the lower 2/3 of the epithelium showed detectable VHR expression and 3+ represented those in which the immunoreactive cells reached the upper epithelial 1/3. For the extent of staining in SCC, normal endocervix, AIS and ADC, 1+ represented samples in which VHR expression was detectable in up to 33% of the epithelium whereas 2+ denoted samples in which 33–66% of the epithelium showed detectable VHR expression and 3+ represented those in which more than 66% of the cells were stained. In order to provide a global score for each case, the results obtained with the two scales were multiplied, yielding a single scale with steps of 0 to 9. The microarrays were scored by 2 independent observers and discrepancies were resolved during a consensus session. To externally validate the staining patterns observed in the TMA, full representative tissue sections of 10 SCC were randomly selected, stained with VHR and scored using the same system as used with the microarray.

### Cell Lines and primary keratinocytes

Five different cell lines derived from cervix cancer were used in this study: HeLa, SiHa and CaSki (all positive for HPV) and C33 and HT3 (HPV negative). Immortalized human foreskin keratinocytes stably transfected with E6 and E7 (E6/E7) were previously described [12] and kindly provided by F. Rosl (Heidelberg, Germany). The cells were grown in DMEM medium (Dulbecco's modified Eagle's medium; ICN; Flow Laboratories) complemented with 10% heated inactivated fetal calf serum (FCS), 30 units/ml of penicillin, 30 μg/ml of streptomycin and 2 mM of L-glutamine.

Primary keratinocytes (KN) were prepared from hysterectomies. Fragments were plunged in a solution containing gentamycin, fungizon and anti-mycoplasm. These fragments were cut in smaller pieces, and then incubated in trypsin-EDTA (Invitrogen) at 37°C under agitation for 1–2 hours. The epithelium was scraped and cells were recovered in FCS. After centrifugation, the cells were resuspended in K-SFM medium (Serum Free Media; Invitrogen) complemented with EGF (0.1 ng/ml), pituitary hormone (20–30 μg/ml) and gentamycin (5 μg/ml).

### Cell lysates and Immunoblotting

Cells were lysed in 20 mM Tris-HCl at pH 7.5, 150 mM NaCl, 5 mM EDTA containing 1% NP-40, 1 mM Na_3_VO_4_, 10 μg/ml aproptinin and leupeptin, 100 μg/ml soybean trypsin inhibitor and 1 mM phenylmethylsulfhonyl fluoride, incubated on ice for 30 min then centrifuged at 20,000 g for 20 min. The proteins were then resolved by SDS-PAGE and transferred onto nitrocellulose membrane. The membranes were immunoblotted with optimal dilutions of monoclonal primary antibodies, followed by an HRP conjugated anti-mouse secondary Ab. The blots were developed by enhanced chemiluminescence (ECL kit, Amersham) according to the manufacturer's instructions.

### RNA preparation and RT-PCR

Total RNA was extracted from primary keratinocytes and the different cell lines using the High Pure RNA Isolation Kit (Roche Diagnostics, Germany) according to the procedures supplied by the manufacturer. Reverse transcription was performed using the RT-PCR kit (Applied Biosystem, Foster City, CA). The PCR reaction was performed using VHR specific primers (5'-ATGTCGGGCTCGTTCGAGCTC-3' and 5'-CTAGGGTTTCAACTTCCCCTC-3') and normalized with HPRT (5'-GTTGGATACAGGCCAGACTTTGTTG-3' and 5'-GATTCAACTTGCGCTCATCTTAGGC-3').

## Results

### VHR expression and localization in cervix biopsies

**Figure 1:**
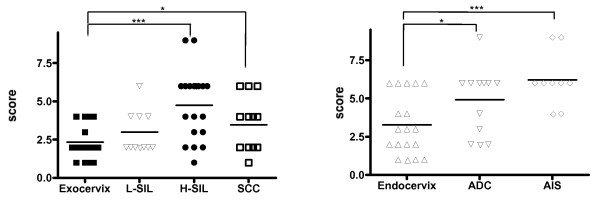
**Semi-quantitative evaluation of the expression of VHR phosphatase in a neoplastic and pre-neoplastic cervical lesions.** A group of 62 hysterectomy or conization specimens showing the various (pre) neoplastic cervical epithelial lesions (L-SIL (n = 11), H-SIL (n = 18), SCC (n = 12), ADC (n = 12) and AIS (n = 9)) and 35 cases of hysterectomy performed for non-cervical pathologies, from patients under 50 years of age was evaluated for the expression of VHR protein using IHC method on a tissue microarray. The score for VHR staining (staining intensity × staining extent) is presented on the y axis. Each point represents one patient and the average expression level is indicated by a bar. NEx, normal exocervix; NEnd, normal endocervix; L-SIL, low-grade SIL; H-SIL, high-grade SIL; ADC, Adenocarcinoma; AIS, adenocarcinoma in situ. * p < 0.05 and ***p < 0.0001.

**Figure 2:**
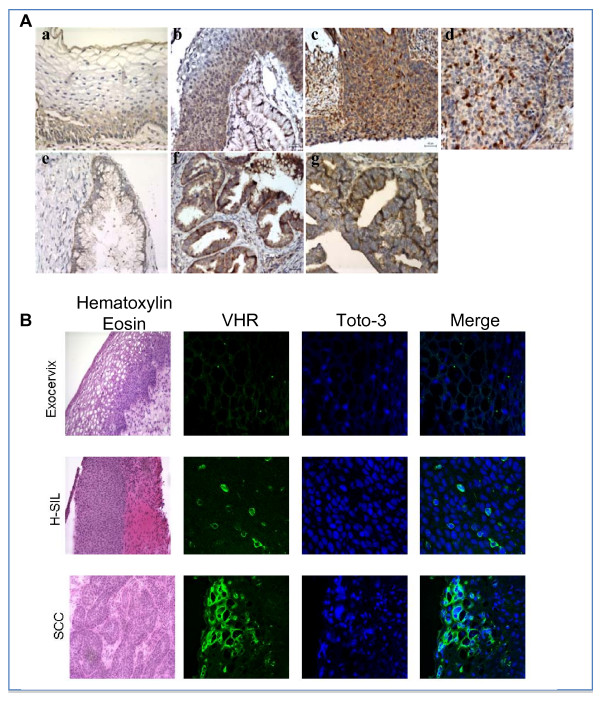
**A/**: Immunohistochemical demonstration of VHR in cervical tissues. a) In normal exocervical tissue, there is a spreaded weak positive staining. b) In L-SIL, the VHR staining is more pronounced but exclusively cytosolic. c) In H-SIL: strong staining of VHR in the cytosol as well as in few nuclei. d) In SCC : strong nuclear staining in an important number of cancerous cells. e) Cervical glandular cells of the normal endocervix show very weak and spreaded staining of VHR. In endocervical adenocarcinoma (f) and AIS (g) there is strong cytoplasmic staining of VHR. Brown color represents VHR specific staining and blue is the staining for nuclei. **B/**: Immunofluorescent staining, under laser scanning confocal miscroscopy, of cervical biopsies showing a low VHR immunoreactivity and absence of nuclear staining in exocervical epithelium and high VHR expression, nuclear and peri-nuclear localisation in H-SIL and SCC. Toto-3 (blue) was used to stain the nuclei and merged to VHR staining (green).

VHR expression at the protein level was studied using a tissue microarray mounted in normal exocervix (*n *= 16), low-grade SILs (*n *= 11), high-grade SILs (*n *= 18), invasive SCCs (*n *= 12), normal endocervix (n = 19), adenocarcinoma (n = 12) and adenocarcinoma in situ (n = 9). Semi-quantitative evaluation of VHR staining is shown in figure 1. The VHR score was statistically higher in H-SILs and SCCs (*p *< 0.0001 and p < 0.05 respectively) compared to normal exocervical epithelium (Fig 1, upper panel). The VHR score in ADC and AIS was also significantly higher in ADC and AIS compared to normal endocervix (p < 0.05 and p < 0.0001 respectively) (Fig. 1 lower panel). The VHR immunoreactivity was very low in the (para) basal cell layers of the normal squamous epithelium (Fig. 2A, panel a) whereas an intense staining was observed in H-SIL and SCC (Fig. 2A, panels c and d). In contrast to normal epithelium, VHR was both nuclear and cytoplasmic in H-SIL and SCC (Fig. 2A, panels c and d). High immunoreactivity of VHR was also observed in ADC and AIS but no nuclear staining was detected in these two categories of cervix cancer (Fig. 2A, panels e, f and g).

In order to confirm the localization differences of VHR in cancer biopsies versus exocervix epithelium, we performed immunofluorescence analysis under laser scanning confocal microscopy. Figure 2B shows that VHR phosphatase is barely detectable and localizes exclusively in the cytosol of the keratinocytes of the normal exocervix. In H-SIL, VHR is highly expressed and has both nuclear and cytoplasmic localization in several cells of the epithelium. However, in SCC, VHR is highly expressed compared to the exocervix of the same patient, with mainly nuclear and perinuclear localization (Fig 2B).

### VHR in cervix cancer cell lines

**Figure 3:**
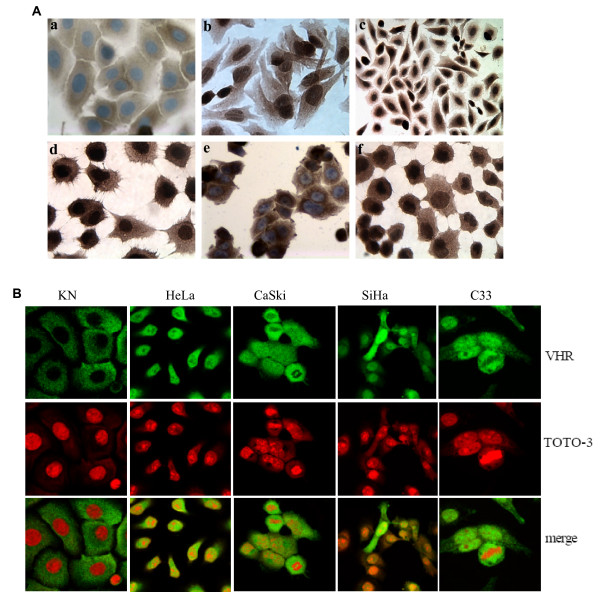
VHR expression and localization in cervix cell lines and primary keratinocytes. **A/ **Immunochemistry of VHR in primary keratinocytes (a), in HPV positive, HeLa (b), SiHa (c) and CaSki (d) and HPV negative C33 (e) and HT3 (f) cell lines. Brown indicates the VHR specific staining and blue represents the hematoxylin staining of nuclei. **B/ **Immunofluorescent staining under confocal microscopy of the primary keratinocytes and cancer cell lines HeLa, CaSki, SiHa and C33. Green indicates the VHR specific staining and Red (TOTO-3) staining for the nuclei.

**Figure 4:**
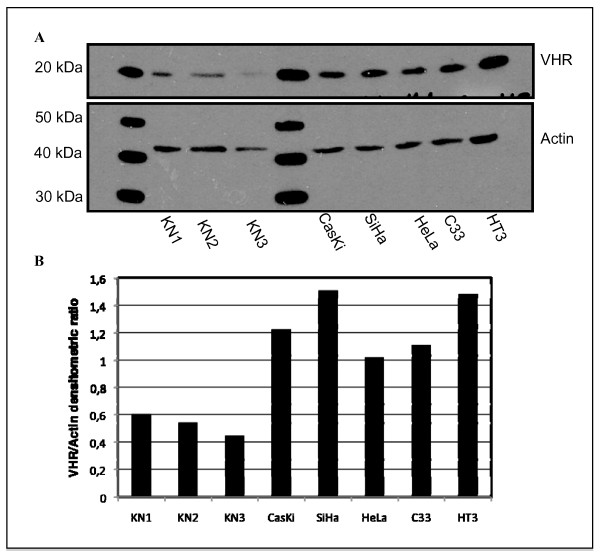
Quantification of VHR protein level. **A**. Representative Western Blot analysis of cell lysates from three different primary keratinocytes (KN) samples and five different cervix cancer cell lines. **B**. Densitometric ratio VHR/Actin for the different cell types of the Western blot shown in panel A. These results are representative of 5 independent experiments.

By immunocytochemistry, the levels of VHR were much higher in the cervix cancer cell lines compared to the primary keratinocytes (Fig. 3A). The presence or absence of HPV in the cells did not affect VHR levels. While VHR was excluded from the nucleus in primary keratinocytes, it was partly nuclear in the cervix cancer cell lines. These observations were confirmed by immunofluorescence staining and confocal microscopy (Fig. 3B). VHR was overexpressed in all the cell lines used compared to primary keratinocytes and was localized in both cytoplasm and nucleus in the cervix cancer cell lines, while it was barely detectable and never nuclear in the primary keratinocytes (Fig. 3B). The degree of VHR overexpression was estimated by immunoblotting and densitometric VHR/Actin ratio to be 1.5 to 2.7 higher in the cervix cancer cells compared to normal cells (Fig. 4).

### VHR half life in cervix cancer cell lines and primary keratinocytes

**Figure 5:**
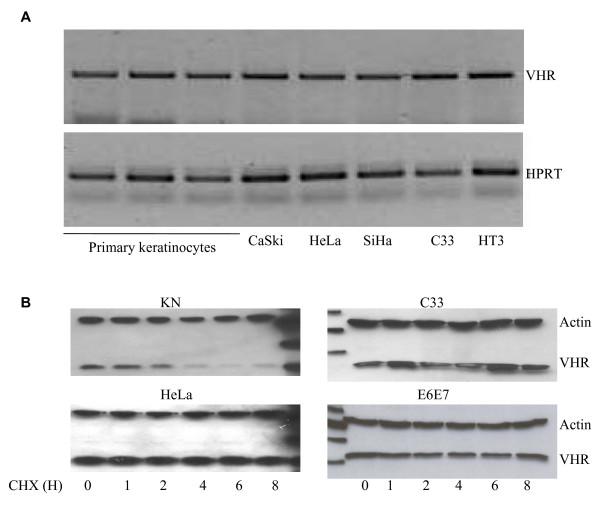
**A/: **Electrophoretic pattern of semi-quantitative RT-PCR products of VHR mRNA performed with primers specific for VHR (upper panel) and the housekeeping gene HPRT (lower panel) (A). mRNA were prepared from primary keratinocytes from 3 different hysterectomies and from 5 different cervix cancer cell lines as indicated on the panels. **B/: **CHX chase analysis by Western blot of primary keratinocytes (KN), HeLa, C33 and E6/E7 cell lines. Cells were treated with CHX (50 μg/ml) and were collected immediately thereafter or after 1, 2, 4, 6 or 8 hours for Western blot analysis using anti-VHR and anti-Actin antibodies.

To elucidate the molecular mechanism responsible for the increased amounts of VHR protein in cervix cancer, we first measured VHR mRNA in the cervix cancer cell lines compared to primary keratinocytes by semi-quantitative RT-PCR. These analyses showed that mRNA levels were indistinguishable between the five cell lines and three different samples of primary keratinocytes derived from hysterectomies (Fig. 5A). These data suggest that the increased amount of VHR protein is not due to increased transcription of DUSP3 locus or stabilization of the VHR mRNA, but more likely caused by increased translation or decreased degradation of VHR protein in the cancer cells.

We examined next the VHR protein turnover by CHX chase and western blot analysis. Primary keratinocytes, HeLa cells, C33 and E6E7 transformed cell lines were incubated with CHX (50 μg/ml) for 1, 2, 4, 6 and 8 hours. Cell lysates were prepared and VHR level versus actin were analyzed by Western blot. After 2 hours of CHX treatment, the level of the expressed VHR decreased by 50% in primary keratinocytes while it did not changed in HeLa, C33 or E6E7 cells up to 8 hours after CHX treatment (Fig 5B). These results demonstrate that VHR half life in primary keratinocytes is shorter than 2 hours and longer than 8 hours in the cancer cells tested.

## Discussion

The development and progression of cervical carcinoma is dependent on both genetic and epigenetic events, including alterations in the cell cycle machinery at various checkpoints. Indeed, cervical carcinoma is associated with aberrant regulation of cyclins D1 and E [13], p16 [14], p21 and p27 [15]. In the present work, we describe that the new discovered cell cycle regulator, the dual-specificity phosphatase VHR [3], is increased at the protein level in five different cervix cancer cell lines positive (HeLa, CaSki and SiHa) or negative (C33 and HT3) for HPV compared to primary keratinocytes prepared from hysterectomies. Thus, VHR expression is not related to the high risk human papillomavirus.

Importantly, the elevated levels of VHR are not an artefact of cell culture. In primary cervix cancer biopsies, VHR is highly expressed in several cells of the epithelium of all the H-SIL analyzed in this study as well as in the SCCs, ADC and AIS. The number of intensely stained cells increased markedly in SCC cases. The staining for VHR and p16 in serial sections showed that all the VHR-positive cells were also positive for p16 (not shown), which is considered as a marker of cervical (pre)cancer cells [12]. This is not surprising since VHR is barely detectable in G1 phase cells, but gradually increases during the progression of the cells to G2/M phase [3]. Thus, VHR, like p16, is a marker of cells in cycle (S, G2 and M phases). These results together suggest that VHR can be considered as a new marker for cancer progression in cervix carcinoma.

We also found that VHR is a cytosolic protein in primary keratinocytes, but localizes both in the cytoplasm and nucleus in cervix cancer cells. VHR does not contain recognizable nuclear localization or nuclear export sequences, but is small enough to passively diffuse into the nucleus. Thus, its exclusion from the nucleus in primary keratinocytes likely is an active process. Preliminary results from our laboratory show that VHR associates with cyclins (unpublished observation), perhaps explaining its retention in the nuclear or cytosolic compartments in normal versus malignant cells. Thus, it appears that VHR location may be connected to cell cycle-dependent transport of cyclins or dysregulation of cyclins in cancer [16].

What may be purpose of elevated VHR in cervix cancer? Based on our previous findings[3,4], we believe that VHR is important for cell cycle progression because it tempers Erk and Jnk during the S and G2/M phases of the cell cycle, where excessive activity of these kinases can trigger cell cycle arrest in G1/S or G2/M. Thus, by elevating the levels of VHR, cancer cells would prevent Erk and Jnk from becoming too active in S through G2, while still allowing them to drive G1 progression. In support of this view, it was recently reported that both Erk and Jnk are less active in cervix cancer cells than in premalignant lesions [18]. Finally, because VHR is regulated during cell cycle progression and because its suppression by RNAi[3] halts cellular proliferation and induces cellular senescence, and, more importantly, because of its overexpression in the cervix cancer, we propose that VHR may be a good target for anticancer therapy. An exciting possibility will be that cervix cancer cells that have adapted to high levels of VHR expression will be sensitive to VHR small inhibitors and that untransformed cells are much less dependent on VHR for proliferation and survival. We have indeed developed novel multidentate small molecule inhibitors of VHR that inhibit its enzymatic activity at nanomolar concentrations *in vitro*, and are active at low micromolar concentrations on several cell lines and primary cells. We have tested these inhibitors on HeLa and primary keratinocytes and demonstrate that they halt HeLa cells proliferation while they have no effect on primary keratinocytes (Tautz L, submitted).

## Conclusion

VHR is an important cell cycle regulator. Loss of this phosphatase causes senescence and prolonged hyperactivation of Erk and Jnk pathways. Together with our new finding reported in this manuscript, these results might ultimately lead in the near future, to a pharmacological approach to inducing senescence in tumor cells, which would be a radical approach to treating cancer.

## Competing interests

The authors declare that they have no competing interests.

## Authors' contributions

RH carried out the IHC, western blotting and RT-PCR studies. PD and JB selected the biopsies from the tumor bank (BTULg) and established the diagnosis. MA performed the tissue microarrays analysis. MM and LT participated in the design of the study. MZ performed the statistical analysis. TM and SR conceived of the study, and participated in its design and coordination and helped to draft the manuscript. All authors read and approved the final manuscript.

## Pre-publication history

The pre-publication history for this paper can be accessed here:


